# Long-distance quantum information transfer with strong coupling hybrid solid system

**DOI:** 10.1038/srep17025

**Published:** 2015-11-20

**Authors:** Feng-Yang Zhang, Xin-Yu Chen, Chong Li, He-Shan Song

**Affiliations:** 1 School of Physics and Materials Engineering, Dalian Nationalities University, Dalian 116600, China; 2 School of Physics and Optoelectronic Technology, Dalian University of Technology, Dalian 116024, China

## Abstract

In this paper, we demonstrate how information can be transferred among the long-distance memory units in a hybrid solid architecture, which consists the nitrogen-vacancy (NV) ensemble acting as the memory unit, the *LC* circuit acting as the transmitter (receiver), and the flux qubit acting as the interface. Numerical simulation demonstrates that the high-fidelity quantum information transfer between memory unit and transmitter (receiver) can be implemented, and this process is robust to both the *LC* circuit decay and NV ensemble spontaneous emission.

Quantum information transfer (QIT) and long-distance quantum communication (LDQC) play an important role in the field of the quantum information^1^. They can transmit quantum information bewteen distant sites. How to realize QIT and LDQC is still an open question. Generally speaking, a good physical system should satisfy two conditions for the QIT and LDQC. First, the system has sufficiently long coherence time, i.e., QIT and LDQC should be achieved before the decoherence happens. Second, the system has a robust channel to avoid the loss of information. As far as we know, there are many proposals for the QIT and LDQC. For example, (i) several optical cavities (microsphere cavities) were linked by fibers (superconducting qubits), and atoms (atomic ensembles, quantum dots, ions, or ionic ensembles) acting as qubits were trapped in each cavity, the deterministic QIT and LDQC were realized with separated qubits^2^,^3^,^4^,^5^,^6^,^7^,^8^. (ii) QIT and LDQC were implemented with separated qubits via the virtual excitation of the data bus to induce the coupling^9^,^10^,^11^,^12^. (iii) Using linear optics devices, the QIT and LDQC were achieved by one photon of an entangled pair in free-space^13^, and so on. Due to optical absorption and channel’s noise, the successful probabilities of the QIT and LDQC will reduce with the increase of the distance. In this paper, we propose a different scheme, which is a good candidate for realizing QIT and LDQC.

On the other hand, among various kinds of solids, the nitrogen vacancy (NV) in diamond has a long coherence time at room temperature^14^ and large capacity of information storage^15^. It is a promising candidate for the storage of the quantum information. In this physical system, the recent experiments have implemented two-qubit conditional quantum gate^16^ and Deutsch-Jozsa algorithm^17^. Another solid system, the superconducting qubits have advantages in design flexibility, large-scale integration, and compatibility to conventional electronics^18^,^19^. And they have shown the superiority in quantum simulation^20^ and generating of the quantum entanglement^21^, *etc*. Thus, the hybrid solid system devices have attracted tremendous attentions, which consist of respect advantages of various physical systems (see^22^ and references therein). Recently, ref. 23 has proposed the magnetic coupling between a superconducting flux qubit and a single NV center can be about 3 orders of magnitude stronger than that associated with stripline resonators. Then, the coherent coupling and information transferred between a flux qubit and a NV ensemble have been implemented^24^,^25^, respectively. In addition, the coupling between single NV center and a superconducting cavity by a flux qubit has been suggested^26^, the strong coupling between a NV ensemble and a transmission-line resonator by a flux qubit was presented^27^, and the short-distance QIT between NV ensembles was proposed^28^.

Motivated by the recent papers^23^,^24^,^25^,^26^,^27^,^28^, here, we elaborate a different proposal to realize QIT and LDQC with simple physical set-ups. As shown in Fig. 1, *Alice* and *Bob* have a same device, respectively, which consists a NV ensemble, a flux qubit, and a *LC* circuit. The NV ensemble acts as the information memory unit, the flux qubit acts as the interface, and the *LC* circuit is a transmitter (receiver) of information. In the large detuning regime, the degrees of freedom of the flux qubit can be eliminated, and we obtain the effective coupling between the NV ensemble and the *LC* circuit. And the entanglement of the two subsystems is induced by a flux qubit. Initially, the information is stored in the memory unit of *Alice*. Then, the information is transferred to transmitter by means of evolution of the system. Through the antenna radiation of the *LC* circuit, the information is transferred in free-space. At distant sites, the information is received by the receiver of *Bob*, then stored in the memory unit. So, the LDQC between two spatially-distant memory units has been achieved.

## Results

### System and Model

**Figure 1:**
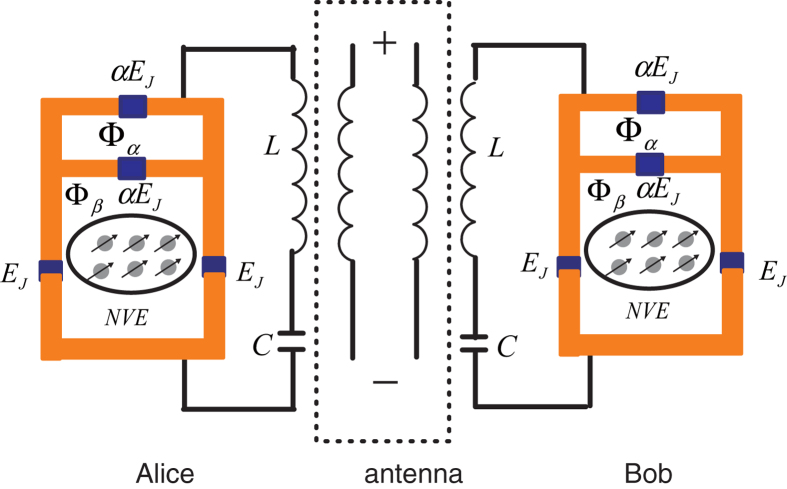
The long-distance quantum communication is realized from Alice to Bob by antenna radiation. Alice and Bob have the same device, respectively, which consists of a NV ensemble, a flux qubit, and a *LC* circuit. The flux qubit consists of four Josephson junctions with the Josephson energies *E*_*J*_ and *αE*_*J*_ (0.5 < *α* < 1). Φ_*α*_ and Φ_*β*_ are the magnetic flux through two loops, respectively. *L* and *C* are the inductance and capacitor of the *LC* circuit, respectively.

The model as shown in Fig. 1. The flux qubit can be described as a two-level system^29^,^30^, the Hamiltonian is (setting *ħ* = 1) 
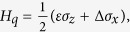
 where *ε*(Φ) = 2*I*_*p*_(Φ − 0.5Φ_0_) is the energy spacing of the two classical current states, *I*_*p*_ is persistent current of the flux qubit, Φ_0_ = *h*/2*e* is the magnetic-flux quantum, Φ = Φ_*α*_/2 + Φ_*β*_ is the external magnetic flux applied in the qubit; Δ is the energy gap between the two states at the degeneracy point; Pauli matrices 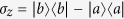 and 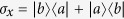 are defined in terms of the classical current where 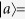 |↻⟩ and 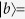 |↺⟩ denote the states with clockwise and counterclockwise currents in the loop. After transformation to the eigenbasis of the flux qubit, the Hamiltonian can be rewritten as 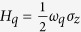, with 
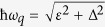
 the energy level separation of the flux qubit.

A NV has an electron spin *S* = 1, with zero-field splitting *D* = 2.88 GHz between the levels *m*_*s*_ = 0 and *m*_*s*_ = ±1^31^. By applying a static magnetic field along to the crystalline axis of diamond, the degeneracy of levels 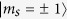 can be removed. The information is encoded in sublevels 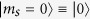 and 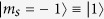 serving as qubit. For the NV ensemble, the ground state is defined as 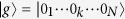 and the excited state is 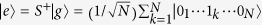 with operator 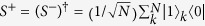
32. Under the large *N* and low excitations conditions, the operators *S*^−^ and *S*^+^ satisfy the bosonic commutation relation, i.e., [*S*^−^, *S*^+^] ≈ 1^33^. Thus, the Hamiltonian of NV ensemble is written as 
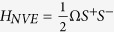
, where Ω = *D* − *g*_*e*_*μ*_*B*_*B*_*z*_ is the energy gap between the ground state sublevels 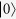 and 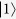 with the magnetic field *B*_*z*_, and *g*_*e*_ and *μ*_*B*_ are the Lande factor and the Bohr magneton, respectively.

The NV ensemble couples to the flux qubit via the magnetic field created. The Hamiltonian for flux qubit coupled to a NV ensemble can be represented by *J*(*S*^+^ + *S*^−^)*σ*_*z*_ with the coupling strength 
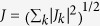
, here *J*_*k*_ is the coupling strength between the flux qubit and NV centers. After a trivial change of basis on the flux qubit and we make a rotating wave approximation, the direct interaction Hamiltonian of the flux qubit and the NV ensemble is *J*(*S*^+^*σ*^−^ + *S*^−^*σ*^+^)^23^.

The *LC* circuit is described by a simple harmonic oscillator Hamiltonian *ωa*^†^*a* with resonance frequency 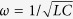, where *a*^†^ and *a* are the plasmon creation and annihilation operators, respectively. In addition, since the interaction between a flux qubit and an *LC* circuit via the mutual inductance *M* has been experimentally realized^34^, the physical features have been widely studied both in theory^35^ and in experiments^36^,^37^. The interaction Hamiltonian is *g*′(*a*^†^ + *a*)*σ*_*z*_ with coupling strength 
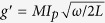
34. At the eigenbasis of the flux qubit, neglecting the small diagonal terms, the interaction Hamiltonian can be written *g*(*aσ*^+^ + *a*^†^*σ*^−^) with effective coupling constant 
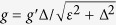
 under the rotating-wave approximation and the condition that Δ > *ε* is satisfied.

According to the above mentions, in the Schrödinger picture, the total Hamiltonian of a single device can be written as




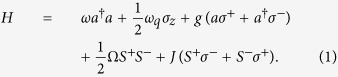




For convenience, the Hamiltonian of the Eq. (1) can be divided into two parts: the free term 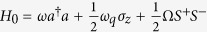 and the interaction term *H*_*I*_ = *g*(*aσ*^+^ + *a*^†^*σ*^−^) + *J*(*S*^+^*σ*^−^ + *S*^−^*σ*^+^). If the conditions 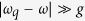 and 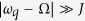 are satisfied (i.e., in the large detuning regime), the effective Hamiltonian of the Eq. (1) is obtained by Fröhlich-Nakajima transformation^38^,^39^. The expression of the effective Hamiltonian is




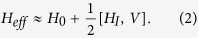




where 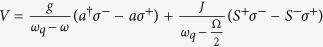 is an anti-Hermitian operator, which satisfies the relation *H*_*I*_ + [*H*_0_, *V*] = 0. The Eq. (2) discards the higher-order terms and only keeps the second-order term.

If the flux qubit is prepared in the ground state 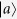 at the initial moment, we can realize the inductive coupling between the *LC* circuit and the NV ensemble by virtual excitation of the flux qubit. So, with the degrees of freedom of the flux qubit are eliminated, the effective Hamiltonian of the hybrid system can be written as




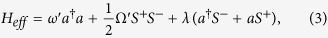




where the parameters 
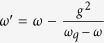
 and 
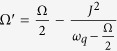
, the last term represents the interaction between the *LC* circuit and the NV ensemble with the effective coupling strength 
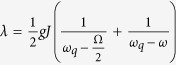
.

### Quantum information transfer

For *n* Fock states in the *LC* circuit, the NV ensemble and *LC* circuit dynamics are completely confined to subspace with basis 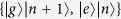. The Hamiltonian (3) can be solved accurately, the eigenstates can be expressed as




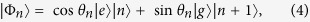







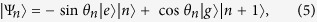




with the parameter 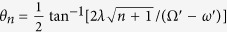, and corresponding to eigenenergies are 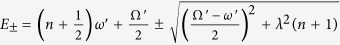. Obviously, the Eq. (4) and (5) represent the entangled states between the *LC* circuit and the NV ensemble. If the information is stored in NV ensemble, we can read out it by measuring quantum states of the *LC* circuit.

In the interaction picture, the Hamiltonian (3) becomes




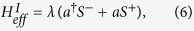




with the resonant interaction Ω′ = *ω*′. If the information is encoded in the NV ensemble at the initial moment, we can realize the information transfer from NV ensemble (memory unit) to *LC* circuit (transmitter), that is, 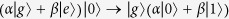 with the evolution time *t*_*p*_ = (2*k* + 1)*π*/2*λ*, (*k* = 0, 1, 2…), where *α* and *β* are the normalized complex numbers. Then, the information of the *LC* circuit can be emitted by the antennary radiation. At the distant receiving terminal, the information is received by another *LC* circuit (receiver), then stored in NV ensemble (memory unit), that is, 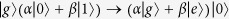. In other word, we realize a LDQC between *Alice* and *Bob* without using data bus (fibers, transmission line resonator, or nanomechanical resonator). Moreover, *Alice* can act as a base station, and *Bob* can act as a user. We can realize the quantum communication from one base station to many users. The channel of our scheme is electromagnetic wave, which has been widely used in the field of communications. We now discuss the dominant noise of the channel due to microwave photons loss. When the electromagnetic wave transmits in free-space, the signal power of the receiver can be written as




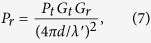




where *P*_*t*_ is the transmitted power, *G*_*t*_ indicates the gain of antenna-transmitter, *G*_*r*_ expresses the gain of antenna-receiver, *d* is the distance between the transmitter and the receiver, and *λ*′ is the wavelength. In order to avoid the loss of the channel, we should shorten the distance *d* between the transmitter and the receiver, or increase the wavelength *λ*′.

## Discussion

**Figure 2:**
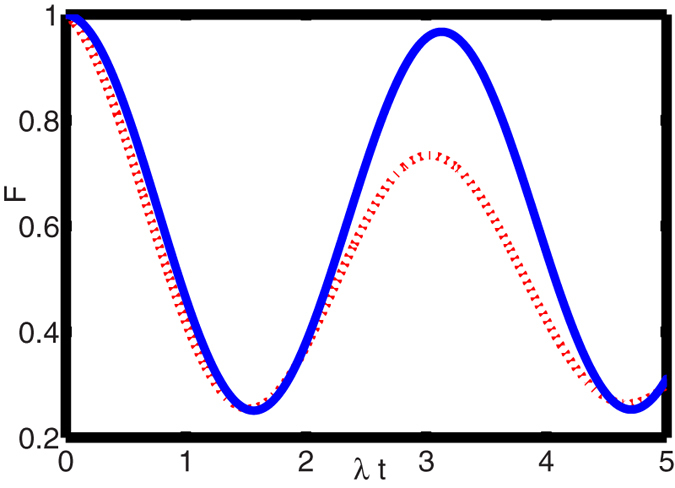
The fidelity of the quantum state transfer from NV ensemble (memory unit) to *LC* circuit (transmitter) versus the dimensionless time *λt* with 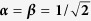. The solid-blue and dot-red lines correspond to *κ* = *γ* = *γ*_*φ*_ = 0.01*λ* and *κ* = *γ* = *γ*_*φ*_ = 0.1*λ*, respectively.

For a really physical system, we should take account of decoherence effects. As *Alice* and *Bob* have the same dissipation mechanism, here, we only discuss the decoherence effects of *Alice*. The flux qubit worked in large detuning regime and prepared in the ground state. The decoherence effective of the flux qubit is omitted. Thus, we only consider the decay of LC circuit, the dephasing and relaxation of NV ensemble. Following the standard quantum theory of the damping, the Markovian master equation is




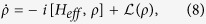




where the Lindblad term 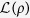 presents the decay of the *LC* circuit and the decoherence of the NV ensemble, and the detailed expression is 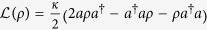 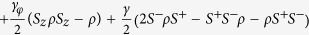 with the decay rate *κ* of the *LC* circuit, and the dephasing rate *γ*_*φ*_ and the relaxation rate *γ* of the NV ensemble. Fidelity is a direct measure to characterize how accurate the information transfer from NV ensemble to *LC* circuit, and its expression is 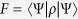, where 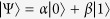 is a target state to be stored in the *LC* circuit. In Fig. 2, we plot the fidelity *F* as a function of the dimensionless time *λt* with the different decay rate *κ*, the dephasing rate *γ*_*φ*_ and the relaxation rate *γ*. This figure shows that the high-fidelity QIT could be achieved in the weak decoherence case.

**Figure 3:**
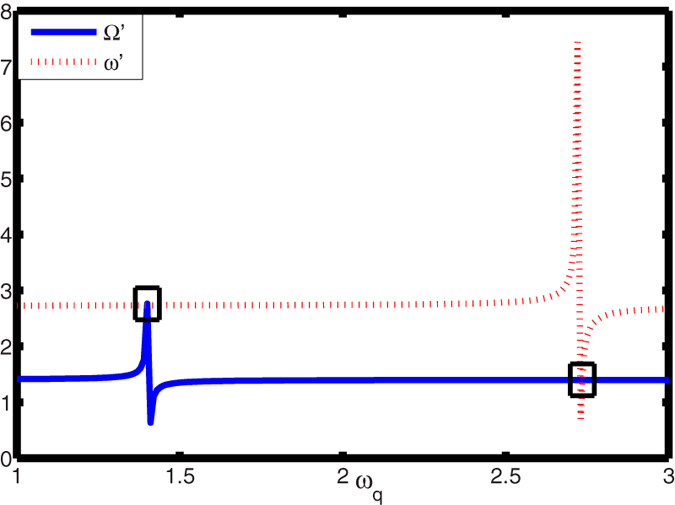
The relation between the Ω′ and the *ω*′ with the change of the *ω*_*q*_. When the *ω*_*g*_ takes certain value (black panes), the resonant condition Ω′ = *ω*′ is achieved.

The experiment^24^ has reported that the coupling strength between flux qubit and NV ensemble is *J* = 70 MHz, the Lande factor is *g*_*e*_ = 2, the Bohr magneton is *μ*_*B*_ = 14 MHz/mT, and the magnetic field is *B*_*z*_ = 2.6 mT. Besides, the strong coupling of a *LC* circuit and a flux qubit has been implemented^37^. In this experiment^37^, the coupling strength between the flux qubit and the *LC* circuit is *g* = 119 MHz, the frequency of the *LC* circuit is *ω* = 2.723 GHz, and the decay rate of the *LC* circuit is *κ* = 0.45 MHz. Through adjusting the frequency *ω*_*q*_ of the flux qubit, the large-detuning between the flux qubit and the *LC* circuit (NV ensemble) can be well satisfied. Also, the resonant condition Ω′ = *ω*′ is satisfied at the proper frequency *ω*_*q*_, see in Fig. 3. According to the above value of parameters, we can estimate the effective coupling strength between NV ensemble and *LC* circuit *λ* ~ 10 MHz. Thus, the strong coupling between NV ensemble and *LC* circuit is realized. So, we can estimate the time *t*_*p*_ ~ 0.16 *μ*s, which is shorter than the decoherence time of the NV ensemble approaching 1 s^40^ and the flux qubits coherence time *T*_2_ ≃ 20 *μ*s^41^.

In summary, we have proposed a hybrid solid architecture, which can realize the strong coupling between a NV ensemble and a *LC* circuit by a flux qubit. We have also shown the high-fidelity quantum information transfer between the NV ensemble and the *LC* circuit. In addition, the LDQC can be implemented using this architecture by the antenna radiation. The proposed architecture opens a way for quantum communication from one base station to many users.

## Additional Information

**How to cite this article**: Zhang, F.-Y. *et al.* Long-distance quantum information transfer with strong coupling hybrid solid system. *Sci. Rep.* **5**, 17025; doi: 10.1038/srep17025 (2015).

## References

[b1] Duan L. M., Lukin M. D., Cirac J. I. & Zoller P. Long-distance quantum communication with atomic ensembles and linear optics. Nature 414, 413 (2001). 11719796 10.1038/35106500

[b2] Cirac J. I., Zoller P., Kimble H. J. & Mabuchi H. Quantum State Transfer and Entanglement Distribution among Distant Nodes in a Quantum Network. Phys. Rev. Lett. 78, 3221 (1997).

[b3] Paternostro M., Kim M. S. & Palma G. M. Non-Local Quantum Gates: a Cavity-Quantum-Electro-Dynamics implementation. J. Mod. Opt. 50, 2075 (2003).

[b4] Serafini A., Mancini S. & Bose S. Distributed Quantum Computation via Optical Fibers. Phys. Rev. Lett. 96, 010503 (2006). 16486430 10.1103/PhysRevLett.96.010503

[b5] Yin Z. Q. & Li F. L. Multiatom and resonant interaction scheme for quantum state transfer and logical gates between two remote cavities via an optical fiber. Phys. Rev. A 75, 012324 (2007).

[b6] Zheng S. B. Virtual-photon-induced quantum phase gates for two distant atoms trapped in separate cavities. Appl. Phys. Lett. 94, 154101 (2009).

[b7] Yang W. L., Hu Y., Yin Z. Q., Deng Z. J. & Feng M. Entanglement of nitrogen-vacancy-center ensembles using transmission line resonators and a superconducting phase qubit. Phys. Rev. A 83, 022302 (2011).

[b8] Yang C. P., Su Q. P. & Nori F. Entanglement generation and quantum information transfer between spatially-separated qubits in different cavities. New J. Phys. 15, 115003 (2013).

[b9] Yang C. P., Zhu S. I. & Han S. Quantum Information Transfer and Entanglement with SQUID Qubits in Cavity QED: A Dark-State Scheme with Tolerance for Nonuniform Device Parameter. Phys. Rev. Lett. 92, 117902 (2004). 15089172 10.1103/PhysRevLett.92.117902

[b10] Blais A. *et al.* Quantum-information processing with circuit quantum electrodynamics. Phys. Rev. A 75, 032329 (2007).

[b11] Cleland A. N. & Geller M. R. Superconducting Qubit Storage and Entanglement with Nanomechanical Resonators. Phys. Rev. Lett. 93, 070501 (2004). 15324218 10.1103/PhysRevLett.93.070501

[b12] Zhang F. Y., Liu B., Chen Z. H., Wu S. L. & Song H. S. Controllable quantum information network with a superconducting system. Ann. Phys. (N.Y.) 346, 103 (2014).

[b13] Pan J. W. *et al.* Multiphoton entanglement and interferometr. Rev. Mod. Phys. 84, 777 (2012), and references therein.

[b14] Balasubramanian G. *et al.* Ultralong spin coherence time in isotopically engineered diamond. Nature Mater. 8, 383 (2009). 19349970 10.1038/nmat2420

[b15] Toyli D. M., Weis C. D., Fuchs G. D., Schenkel T. & Awschalom D. D. Quantum control and nanoscale placement of single spins in diamond. Nano. Lett. 10, 3168 (2010). 20698632 10.1021/nl102066q

[b16] Jelezko F. *et al.* Observation of Coherent Oscillation of a Single Nuclear Spin and Realization of a Two-Qubit Conditional Quantum Gate. Phys. Rev. Lett. 93, 130501 (2004). 15524692 10.1103/PhysRevLett.93.130501

[b17] Shi F. *et al.* Room-Temperature Implementation of the Deutsch-Jozsa Algorithm with a Single Electronic Spin in Diamon. Phys. Rev. Lett. 105, 040504 (2010). 20867828 10.1103/PhysRevLett.105.040504

[b18] Makhlin Y., Schön G. & Shnirman A. Quantum-state engineering with Josephson-junction devices. Rev. Mod. Phys. 73, 357 (2001).

[b19] You J. Q. & Nori F. Atomic physics and quantum optics using superconducting circuits. Nature 474, 589 (2011). 21720362 10.1038/nature10122

[b20] Underwood D., Shanks W., Koch J. & Houck A. A. Low-disorder microwave cavity lattices for quantum simulation with photons. Phys. Rev. A 86, 023837 (2012).

[b21] Wang H. *et al.* Deterministic Entanglement of Photons in Two Superconducting Microwave Resonator. Phys. Rev. Lett. 106, 060401 (2011). 21405445 10.1103/PhysRevLett.106.060401

[b22] Xiang Z. L., Ashhab S., You J. Q. & Nori F. Hybrid quantum circuits: Superconducting circuits interacting with other quantum systems. Rev. Mod. Phys. 85, 623 (2013).

[b23] Marcos D. *et al.* Coupling Nitrogen-Vacancy Centers in Diamond to Superconducting Flux Qubits. Phys. Rev. Lett. 105, 210501 (2010). 21231275 10.1103/PhysRevLett.105.210501

[b24] Zhu X. *et al.* Coherent coupling of a superconducting flux qubit to an electron spin ensemble in diamond. Nature (London) 478, 221 (2011). 21993757 10.1038/nature10462

[b25] Saito S. *et al.* Towards Realizing a Quantum Memory for a Superconducting Qubit: Storage and Retrieval of Quantum States. Phys. Rev. Lett. 111, 107008 (2013). 25166702 10.1103/PhysRevLett.111.107008

[b26] Twamley J. & Barrett S. D. Superconducting cavity bus for single nitrogen-vacancy defect centers in diamond. Phys. Rev. B 81, 241202 (R) (2010).

[b27] Xiang Z. L., Lü X. Y., Lie T. F., You J. Q. & Nori F. Hybrid quantum circuit consisting of a superconducting flux qubit coupled to a spin ensemble and a transmission-line resonator. Phys. Rev. B 87, 144516 (2013).

[b28] Zhang F. Y., Yang C. P. & Song H. S. Scalable quantum information transfer between nitrogen-vacancy-center ensembles. Ann. Phys. (N.Y.) 355, 170 (2015).

[b29] Orlando T. P. *et al.* Superconducting persistent-current qubit. Phys. Rev. B 60, 15398 (1999).

[b30] Mooij J. E. *et al.* Josephson Persistent-Current Qubit. Science 285, 1036 (1999). 10446043 10.1126/science.285.5430.1036

[b31] Wrechtrup, J. & Jelezko F. Processing quantum information in diamond. J. Phys.: Condens. Matter 18, S807 (2006).

[b32] Taylor J. M., Marcus C. M. & Lukin M. D. Long-Lived Memory for Mesoscopic Quantum Bits. Phys. Rev. Lett. 90, 206803 (2003). 12785914 10.1103/PhysRevLett.90.206803

[b33] Sun C. P., Li Y. & Liu X. F. Quasi-Spin-Wave Quantum Memories with a Dynamical Symmetry. Phys. Rev. Lett. 91, 147903 (2003). 14611556 10.1103/PhysRevLett.91.147903

[b34] Johansson J. *et al.* Vacuum Rabi Oscillations in a Macroscopic Superconducting Qubit LC Oscillator System. Phys. Rev. Lett. 96, 127006 (2006). 16605950 10.1103/PhysRevLett.96.127006

[b35] Liu Y. X., Sun C. P. & Nori F. Scalable superconducting qubit circuits using dressed state. Phys. Rev. A 74, 052321 (2006).

[b36] Koch R. H. *et al.* Experimental Demonstration of an Oscillator Stabilized Josephson Flux Qubit. Phys. Rev. Lett. 96, 127001 (2006). 16605945 10.1103/PhysRevLett.96.127001

[b37] Fedorov A. *et al.* Strong Coupling of a Quantum Oscillator to a Flux Qubit at Its Symmetry Point. Phys. Rev. Lett. 105, 060503 (2010). 20867965 10.1103/PhysRevLett.105.060503

[b38] Fröhlich H. Theory of the Superconducting State. I. The Ground State at the Absolute Zero of Temperature. Phys. Rev. 79, 845 (1950).

[b39] Nakajima S. Perturbation theory in statistical mechanics. Adv. Phys. 4, 363 (1953).

[b40] Bar-Gill N., Pham L. M., Jarmola A., Budker D. & Walsworth R. L. Solid-state electronic spin coherence time approaching one second. Nature Commun. 4, 1743 (2013). 23612284 10.1038/ncomms2771

[b41] Bylander J. *et al.* Noise spectroscopy through dynamical decoupling with a superconducting flux qubit. Nat. Phys. 7, 565 (2011).

